# NeuN As a Neuronal Nuclear Antigen and Neuron Differentiation Marker

**Published:** 2015

**Authors:** V. V. Gusel’nikova, D. E. Korzhevskiy

**Affiliations:** Federal State Budgetary Scientific Institution “Institute of Experimental Medicine”, akad. Pavlov str., 12, St. Petersburg, 197376, Russia

**Keywords:** NeuN nuclear protein, neuron specific marker, neurons

## Abstract

The NeuN protein is localized in nuclei and perinuclear cytoplasm of most of the neurons in the central nervous system of mammals. Monoclonal antibodies to the NeuN protein have been actively used in the immunohistochemical research of neuronal differentiation to assess the functional state of neurons in norm and pathology for more than 20 years. Recently, NeuN antibodies have begun to be applied in the differential morphological diagnosis of cancer. However, the structure of the protein, which can be revealed by antibodies to NeuN, remained unknown until recently, and the functions of the protein are still not fully clear. In the present mini-review, data on NeuN accumulated so far are summarized and analyzed. Data on the structure and properties of the protein, its isoforms, intracellular localization, and hypothesized functions are reported. The application field of immunocytochemical detection of NeuN in scientific and clinical studies, as well as the difficulties in the interpretation of the obtained experimental data and their possible causes, is described in details.

## INTRODUCTION

Studies of the neural tissue proteome and immunocytochemical studies of nervous system organs have established that neurons contain a number of specific proteins, whose appearance in postmitotic cells is indicative of their neuronal differentiation. Some of these proteins are characteristic of only a number of specific neuronal types. Thus, tyrosine hydroxylase, an enzyme involved in the synthesis of catecholamines, can be detected in the population of catecholaminergic neurons and monoenzyme neurons involved in the synthesis of catecholamines [1, 2], while choline acetyltransferase allows one to label cholinergic neurons [3]. Other specific proteins are present in the vast majority of neurons. One of them is the neuronal nuclear protein NeuN, which is often used as a marker of postmitotic neurons due to some of its properties (primarily nuclear localization) [4-7]. Monoclonal antibodies to the NeuN protein have been actively used in immunohistochemical studies of neuronal differentiation to assess the functional state of neurons in norm and pathology for more than 20 years. Currently, they are also used in the differential morphological diagnosis of cancer [8-10]. However, the structure of the protein, which can be revealed by antibodies to NeuN, remained unknown until recently, and the functions of this protein remain not entirely clear.

The purpose of our study was to summarize and analyze data on the NeuN protein accumulated to date. Data on the structure and properties of the protein, its isoforms, intracellular localization, and hypothesized functions are reported. The application field of immunocytochemical detection of NeuN in scientific and clinical studies, as well as the difficulties in the interpretation of the obtained experimental data and their possible causes, is described in details.

**NeuN protein expression in nervous system cells**

The neuronal nuclear protein (NeuN) was discovered in 1992, when a research team managed to obtain monoclonal antibodies (A60 clone) to this hitherto unknown nuclear protein [11]. Comprehensive immunohistochemical (IHC) analyses have shown that the expression of the NeuN protein throughout the whole ontogeny is exclusively associated with the nervous tissue. This marker has not been detected in tissues other than nervous ones. Moreover, the protein has never been detected in glial cells, which suggests it is a specific neuronal marker. Subsequent studies have shown that anti-NeuN antibodies can identify most types of neurons in the whole nervous system with rare exceptions. Thus, Cajal-Retzius cells in the neocortex, some cerebellar cells (including Purkinje cells), inferior olive neurons, cells of the inner nuclear layer of the retina, γ-motor neurons in the spinal cord, and ganglion cells of the sympathetic chain are not immunohistochemically stained with antibodies to NeuN. There also exist conflicting reports on the expression of NeuN cells in the substantia nigra cells of the brain [12-14]. The causes behind the lack of NeuN immunoreactivity in certain types of neurons have not been established. For example, inferior olive neurons are believed to share a common origin with neurons in the base of the pons, but the latter demonstrate high NeuN immunoreactivity for almost the entire ontogeny, whereas the inferior olive neurons are NeuN-immunonegative in both the fetal and postnatal life periods [15]. Thus, NeuN immunoreactivity apparently reflects some other side of cell biology, rather than a close relationship in embryonic neurohistogenesis.

It is believed that NeuN emerges during early embryogenesis in postmitotic neuroblasts and remains in differentiating and terminally differentiated neurons throughout the whole subsequent ontogeny. Antibody binding to the NeuN protein is predominantly associated with cell nuclei and, to a lesser extent, with the perinuclear cytoplasm [11]. It was shown that two hypothesized isoforms of the NeuN protein (46 and 48 kDa) are present in both locations, but differ in their relative concentration in the nucleus and cytoplasm. Thus, both isoforms of the protein are approximately equally represented in the nucleus, and isoform with a molecular mass of 46 kDa only occasionally predominates, while the isoform with a molecular mass of 48 kDa always predominates in the cytoplasm. It is believed that NeuN isoforms differ in a short amino acid sequence, which is responsible for the localization of the different variants of this protein in the cell [16].

In the nucleus, NeuN is primarily located in the areas with low chromatin density, and it is absent in areas with dense packing of DNA [16]. Most of the intranuclear NeuN is bound to the nuclear matrix [17]. The results of chromatographic analysis of cerebral nuclear proteins demonstrate the ability of the NeuN protein to bind to DNA [11]. It remains not fully clear how specific this binding is and whether NeuN binds to DNA* in vivo*. The nuclear localization of the NeuN protein, its DNA binding properties that were demonstrated* in vitro*, as well as its solubility suggest that NeuN is a neurospecific regulatory molecule functioning at the level of the cell nucleus [11]. More recent studies [17] have confirmed the validity of this assumption. However, the capability of binding to RNA rather than DNA is currently considered to be a more important property of NeuN [18]. Nevertheless, the fact that the expression of NeuN is associated with neuronal differentiation and persists throughout the whole cell life can be an indication that NeuN is a permanent regulator of the general presentation of the neuronal phenotype. In this case, the lack of NeuN expression in certain neuronal populations implies the presence of alternative, but functionally similar to NeuN regulatory molecules in these cells. This assumption is consistent with the general idea that a variety of alternative regulatory mechanisms, providing comprehensive control of the differentiation processes of nerve elements and formation of nervous system organs, should be present in such a complex system as the nervous system of vertebrates.

The accumulated so far experimental data provide evidence that the intensity of the immunocytochemical reactions for NeuN in the nucleus and cytoplasm may vary both within the same type of neurons and between different types of neurons. Thus, an investigation in NeuN distribution in the substantia nigra cells of the rat brain revealed that it is poorly expressed in some neurons, while in other neurons it is completely absent [12]. In humans, the population of neurons of the substantia nigra is also heterogeneous in terms of NeuN distribution. Both weakly immunopositive and immunonegative cells have been detected [13]. The neurons of the substantia nigra differ both in their ability to be stained in an immunohistochemical reaction for NeuN and neuromelanin content in their cytoplasm. Neurons that contain neuromelanin and the NeuN protein; neurons that contain neuromelanin, but give a negative reaction for the NeuN protein; and neurons that do not contain neuromelanin, but contain NeuN were detected. Interestingly, the concentration of the NeuN protein in substantia nigra neurons is significantly lower than that in the neurons of the red nucleus located anatomically close to the substantia nigra and other areas of the human brain [13].

Although NeuN expression in substantia nigra neurons has been convincingly determined in laboratory animals and humans, we can state that, in general, no clear correlation between the intensity of NeuN immunoreactivity and a certain type of neurons has been established. Obviously, the differences in the intensity of the reaction for NeuN reflect the differences in the expression of this protein in a cell, which are associated with both the constitutive characteristics of the neuron and its functional state. Thus, the intensity of the immunocytochemical reaction for NeuN consistently varies during the stimulation of primary neuronal culture cells [19]. Injuries to the nervous system can affect the expression of the NeuN protein in the cell in various ways. For example, axonal injury leads to an almost complete loss of NeuN immunoreactivity in motoneurons of the facial nerve nucleus, while a transection of the rubrospinal tract only leads to a minor reduction in NeuN immunoreactivity in red nucleus neurons [20]. In the latter case, the less pronounced changes may be due to a more distal transection of axons having a sufficient amount of collaterals.

The complexity associated with interpreting the results of immunohistochemical staining for the NeuN protein is associated with the fact that a negative result of the reaction may be due to several reasons. On the one hand, this may be due to the absence of NeuN protein expression in a cell or protein synthesis in such a small amount that it cannot be detected by immunohistochemistry. On the other hand, there is experimental evidence of the influence of NeuN protein phosphorylation on its ability to bind known antibodies to NeuN [16]. It has been shown that there are seven post-translational modifications (forms) of the NeuN protein characterized by varying degrees of phosphorylation. Enzymatic dephosphorylation experiments demonstrated that antibodies to the NeuN protein (clone A60) recognize only phosphorylated forms of the protein, and that at least one phosphate group in the NeuN molecule is required for proper formation of the antigenic determinant recognized by these antibodies [16]. Later on, it was suggested that the epitope for antibody binding to a non-phosphorylated NeuN protein is involved in protein-protein interactions, and, therefore, it is masked and incapable of binding to antibodies [21]. This hypothesis is indirectly confirmed by the fact that the aforementioned epitope has proline-rich amino acid sequences that are considered to be the main actors in protein-protein interactions in the cell [22].

**The structure and properties of NeuN/Fox-3 protein**

For a long time, the nucleotide sequence encoding the NeuN protein remained unknown. In 2009, a research team in the USA [23] carried out a mass spectrometry analysis of peptides derived from trypsinization of the protein, reacting with antibodies to NeuN (clone A60). As a result, the primary structure of the Fox-3 protein was established. The protein consists of 374 amino acids and can exist in four isoforms generated by alternative splicing of the mRNA. Kim *et al*. [23] demonstrated that the protein that reacts with antibodies to Fox-3 interacts with tissue antigens in the same way as known anti-NeuN antibodies. The character of intracellular structure staining upon reaction with anti-Fox-3 antibodies is completely identical to the results of an immunocytochemical reaction for NeuN. It was also shown that expression of the NeuN protein is tapered when using small hairpin RNAs (shRNA) against Fox-3. Finally, it turned out that Fox-3, similarly to NeuN, is expressed only in the nervous tissue. Based on these experimental data, the authors concluded that the NeuN protein is a product of the *Fox-3 *gene*, *which belongs to the *Fox-1 *gene family*. *This work [23] was performed at a high methodological level, using modern molecular- genetic, cytological, and histological methods and made a significant contribution to our understanding of the molecular nature of the antigenic determinant that binds A60 antibodies. Most authors, when investigating NeuN, share Kim’s opinion on the identity of the NeuN antigen and Fox-3 protein, as evidenced by the numerous references to this work (89 references by December 2014) in the articles refereed in databases belonging to the Web of Science service (Thomson Reuters).

Importantly, the same research team [23] reported the detected cross-reactivity of A60 antibodies to the NeuN protein with synapsin I, a member of the neuron- specific phosphoprotein family associated with synaptic vesicles, which play a role in the synaptogenesis and modulation of neurotransmitter secretion. Cross-reactivity is apparently due to the presence of a fragment consisting of 14 homologous amino acid residues in Fox-3 and synapsin I. A part of this fragment is probably involved in the formation of the epitope recognized by A60 antibodies. Importantly, the cross-reactivity of the epitopes of synapsin and NeuN was observed only when using the immunoblotting method, while anti-NeuN antibodies did not bind to synapsin I in an immunocytochemical study on paraffin sections. This may be associated with both the masking of the antigenic determinant due to fixation in formaldehyde and the pouring of paraffin over the material and low affinity of anti-NeuN antibodies to the synapsin I epitope, which is compensated by the high concentration of synapsin in the material under study in immunoblotting [21, 23].

Sequencing and identification of the gene encoding the NeuN protein naturally led to an investigation of the NeuN/Fox-3 functions in nervous system cells. It was shown that this protein plays a role in neurospecific alternative splicing [24]. Subsequently, it has been experimentally established that regulated NeuN/Fox-3 splicing greatly contributes to the regulation of neuron differentiation in the nervous system of vertebrates [25]. In this regard, it is suggested that the functions of the Fox-3 protein in a cell should be taken into account when using NeuN-immunostaining as a convenient neuronal marker. [21].

**Using NeuN protein as a neuromarker**

Although the structure of the antigenic determinant that binds A60 antibodies and the conditions of this binding are not fully understood, antibodies to the NeuN protein are widely used in scientific research and in histopathologic diagnosis. Thus, during the last decade, the NeuN protein has been used as a universal neuron-specific marker for studying the differentiation of stem cells [7, 26-28]. The presence of some specific marker proteins, whose immunocytochemical detection allows for selective identification of cells belonging to the nervous system tissues, in postmitotic cells provides evidence of neuronal differentiation. Such proteins include, for example, β-tubulin III, MAP-2, doublecortin, synaptophysin, neurofilament proteins, neuron-specific enolase, the neural cell adhesion molecule, as well as the neurotransmitter synthesis enzymes (tyrosine hydroxylase, choline acetyl transferase), etc. [28 29]. The use of the NeuN protein as a neuronal differentiation marker has several advantages. Firstly, the NeuN protein is expressed exclusively in the nervous tissue, while other neuronal differentiation marker proteins are also found in other cells. For example, MAP-2 is expressed not only in neurons, but also in skeletal muscles, epithelial cells, etc. Astrocytes also give positive immunocytochemical reaction for neuron-specific enolase, and synaptophysin was found not only in neurons, but also in neuroendocrine cells [29]. Secondly, NeuN is not found in immature neural progenitor cells as long as they are not out of the cell cycle [15, 19, 30]. In this context, some markers are less convenient, as they detect both mature neurons and undifferentiated neuroepithelial cells (MAP-2), or only nerve cells at the late stages of differentiation (neurotransmitters synthesis marker enzymes) [31]. Finally, the NeuN protein is the only one of these markers whose expression is primarily associated with the cell nucleus. In connection to this, detection of this protein, in contrast to cytoplasmic markers, does not depend on a small volume of cytoplasm, which is typical of neuroblasts and small neurons. In addition, nuclear localization of this marker allows one to obtain discrete stained structures of specimens, which are available for binarization (image processing procedures required during the automated quantitative analysis of the objects) when being photographed.

The reaction for NeuN is also used in pathohistological diagnosis in neurooncology [9, 31]. There is evidence of NeuN expression in some cells of differentiated neuronal tumors (neurocytomas, gangliocytomas, medulloblastomas) [30, 31]. For example, Wolf *et al*. [30] revealed NeuN immunoreactivity in the nuclei of some ganglioma cells and absence of such immunoreactivity in oligodendroglial cells, which can be used for the differential morphologic diagnosis of cancer. Inclusion of this marker into an antibody panel used for the diagnosis of neurocytomas can result in improved reliability of diagnostics and differential diagnosis, at least in the case of central nervous system neuroblastomas [31].

Although NeuN is considered to be a convenient marker of postmitotic neurons and differentiated cells of neurogenic tumors, one should be careful when using the protein for identifying neural cells *in vitro*. As shown by Darlington *et al*. [32], NeuN immunoreactivity is present in the primary cell cultures of the murine, rat and human brain. But not only neurons are NeuN-immunopositive. It has been shown that some NeuN-immunopositive cells in these cultures express the glial fibrillary acidic protein (GFAP), an astrocyte marker. Moreover, NeuN is expressed by all GFAP-positive cells. The identified NeuN+/GFAP+ cells demonstrate astrocyte morphology, do not proliferate (according to the results of BrdU labeling), and demonstrate no expression of other neuronal markers. Based on these data, we suggested that NeuN+/ GFAP+ cells identified *in vitro *are astrocytes rather than partially differentiated neuronal precursors at the stage of the beginning of synthesis of neuron-specific proteins as might have been expected as an alternative. Apart from astrocytes, one of the fibroblast cell lines (3T3) proved to be immunoreactive to NeuN* in vitro*. The reason for the NeuN immunoreactivity of non-neuronal cells observed in cultures remains not fully understood. When working with paraffin sections, it was found that NeuN immunoreactivity is affected by some methodological techniques [33-36]. It was noted that long-term fixation in formalin (for several months or years) reduces NeuN immunoreactivity as compared to the level observed after fixation of the same materials for several days or weeks. Furthermore, thermal unmasking of the antigen is usually required for A60 antibody binding [15, 36]. At the same time, decalcification of the objects in a formic acid solution does not lead to a deterioration of the reaction for NeuN [35]. Obviously, NeuN immunostaining involves specific protocols that are standardized for use with paraffin sections [29, 37, 38] but are likely to require further improvement and standardization in the case of *in vitro *studies.

Another application of anti-NeuN antibodies is associated with the identification of pathological changes in existing neuronal populations. Various pathological processes accompanied by a weakening or disappearance of NeuN immunoreactivity in neurons have been reported in several studies. Thus, complete disappearance of NeuN immunohistochemical staining of neuronal nuclei and cytoplasm at the area of ischemic damage to the striatum in a rat brain [39, 40] has been noticed. Termination of NeuN protein synthesis by certain striatal neurons in Huntington’s disease has also been observed [41]. It has been shown that the NeuN nuclear protein disappears from damaged or dying pyramidal neurons in the hippocampus [42]. A decrease in NeuN immunoreactivity in hypoxia and brain injury was also reported [43-45].

It is important to note that in some studies the loss of NeuN immunoreactivity was explained by neuronal death. Thus, Davoli *et al*. [44] compared NeuN-immunostaining with TUNEL staining in ischemia and found that NeuN immunoreactivity was significantly reduced 24 hours after exposure, which correlates with the increase in the number of apoptotic cells (detected by TUNEL). Based on these data, it was suggested that the decrease in NeuN immunostaining is associated with neuronal death in the damaged area of the brain. On the other hand, it was subsequently shown that the loss of NeuN-staining is not always associated with neuronal death and may be effected by other agents; for example, temporarily suspended synthesis of this protein by neurons due to damage (but without viability loss). When using a moderate ischemia model (30 min ischemia), it was found that neurons lose NeuN immunoreactivity 6 h after exposure, while retaining the integrity of the cell and intact nucleus; i.e., they do not exhibit typical signs of cell death [45]. According to the authors, the loss of immunoreactivity in this case is associated with the loss of the antigen’s ability to bind anti-NeuN antibodies, rather than a reduction in NeuN protein synthesis in neurons. In contrast, in the case of axotomy, a sharp decrease in the amount of the NeuN protein in neurons was shown [20]. Therefore, the loss of neuronal NeuN immunoreactivity is indicative of damage, but it cannot be definitive evidence of neuronal death (expected or actual). This fact should be borne in mind when interpreting the results of quantitative immunohistochemical studies.

## CONCLUSIONS

In conclusion, despite the many years of intensive studies of the NeuN protein, a number of issues related to its structure and functions remain open. Thus, antigenic determinants that bind anti-NeuN antibodies and the conditions required for effective interaction between antibodies and the antigen both *in vivo *and *in vitro *remain poorly studied. The entire range of functions of the NeuN protein in cells has not been determined. It is unclear what processes in cells lead to the changes in the intensity of the reaction for NeuN/Fox-3 or loss of NeuN immunoreactivity, as well as post-translational modifications in this protein, which are observed in some cases. Despite this, NeuN has been successfully used for more than 20 years as a reliable marker of postmitotic neurons in studies of neuronal differentiation and in the assessment of neuronal status both in norm and pathology. In recent years, there has been an increase in the number of studies aimed at investigating the properties of the NeuN/Fox-3 protein. New data should deepen our understanding of the structure and functions of this protein and facilitate the objective interpretation of research results using antibodies to the NeuN protein.

## Abbreviations

IHC: immunohistochemical analysis
NeuN: neuronal nuclear protein
shRNA: small hairpin RNA
MAP-2: microtubule-associated protein 2
GFAP: Glial Fibrillary Acidic Protein
TUNEL: Terminal Deoxynucleotidyl Transferase-Mediated dUTP (2’-Deoxyuridine 5’-Triphosphate) Nick-End Labeling
BrdU: 5-bromo-2’-deoxyuridine

## References

[1] Ugrumov M.V. (2009). J Chem Neuroanat..

[2] Ugrumov M., Taxi J., Pronina T., Kurina A., Sorokin A., Sapronova A., Calas A. (2014). Neuroscience..

[3] Korzhevskii D. E., Grigoriev I. P., Kirik O. V., Sukhorukova E. G., Alekseyeva O. S. (2014). Journal of Evolutionary Biochemistry and Physiology..

[4] Korzhevskii D.E., Petrova E.S., Kirik O.V., Otellin V.A. (2009). Neurosci Behav Physiol..

[5] Petrova E. S., Isaeva E. N., Korzhevskii D. E. (2014). Neuroscience and Behavioral Physiology..

[6] Petrova E.S., Isaeva E.N., Korzhevskii D.E. (2014). Bull of Exp Biol Med..

[7] Verdiev B.I., Poltavtseva R.A., Podgornyi O.V., Marei M.V., Zinovyeva R.D., Sukhikh G.T., Aleksandrova M.A. (2009). Bull Exp Biol Med..

[8] Chan M.H., Kleinschmidt-Demasters B.K., Donson A.M., Birks D.K., Foreman N.K., Rush S.Z. (2012). Pediatr Blood Cancer..

[9] You H., Kim Y.I., Im S.Y., Suh-Kim H., Paek S.H., Park S.H., Kim D.G., Jung H.W. (2005). J Neurooncol..

[10] Hagel C., Treszl A., Fehlert J., Harder J., von Haxthausen F., Kern M., von Bueren A.O., Kordes U. (2013). J Neurooncol..

[11] Mullen R.J., Buck C.R., Smith A.M. (1992). Development..

[12] Cannon J.R., Greenamyre J.T. (2009). Neurosci. Lett..

[13] Sukhorukova E. G. (2014). Neuroscience and Behavioral Physiology..

[14] Kumar S.S., Buckmaster P.S. (2007). Brain Research..

[15] Sarnat H.B., Nochlin D., Born D.E. (1998). Brain Dev..

[16] Lind D., Franken S., Kappler J., Jankowski J., Schilling K. (2005). J Neurosci Res..

[17] Dent M.A., Segura-Anaya E., Alva-Medina J., Aranda-Anzaldo A. (2010). FEBS Lett..

[18] Darnell R.B. (2013). Annu Rev Neurosci..

[19] Weyer A., Schilling K. (2003). J Neurosci Res..

[20] McPhail L.T., McBride C.B., McGraw J., Steeves J.D., Tetzlaff W. (2004). Exp Neurol..

[21] Maxeiner S., Glassmann A., Kao H.T., Schilling K. (2014). Histochem Cell Biol..

[22] Williamson M.P. (1994). Biochem J..

[23] Kim K.K., Adelstein R.S., Kawamoto S. (2009). J Biol Chem..

[24] Kim K.K., Kim Y.C., Adelstein R.S., Kawamoto S. (2011). Nucleic Acids Res..

[25] Kim K.K., Nam J., Mukouyama Y.S., Kawamoto S. (2013). J Cell Biol..

[26] Hess D.C., Hill W.D., Martin-Studdard A., Carroll J., Brailer J., Carothers J. (2002). Stroke..

[27] Tanvig M., Blaabjerg M., Andersen R.K., Villa A., Rosager A.M., Poulsen F.R., Martinez-Serrano A., Zimmer J., Meyer M. (2009). Brain Res..

[28] Korzhevskii D.E., Petrova E.S., Kirik O.V., Beznin G.V., Sukhorukova E. G. (2010). Cell Transplantology and Tissue Engineering..

[29] Korzhevskii D.E., Kirik O.V., Petrova E.S., Karpenko M.N., Grigor’ev I.P., Sukhorukova E.G., Kolos E. A. (2014). Theoretical bases and practical application of the immunohistochemical methods. SPb: SpecLit, 2014.

[30] Wolf H.K., Buslei R., Schmidt-Kastner R., Schmidt-Kastner P.K., Pietsch T., Wiestler O.D., Blümcke I. (1996). J Histochem Cytochem..

[31] Soylemezoglu F., Onder S., Tezel G. G., Berker M. (2003). Pathol Res Pract..

[32] Darlington P.J., Goldman J. S., Cui Q.L., Antel J.P., Kennedy T.E. (2008). J Neurochem..

[33] Korzhevskii D.E., Gilerovich E.G., Zin’kova N.N., Grigor’ev I.P., Otellin V.A. (2006). Neurosci Behav Physiol..

[34] Korzhevskii D. E., Sukhorukova E. G., Gilerovich E. G., Petrova E. S., Kirik O. V., Grigor’ev I. P. (2014). Neuroscience and Behavioral Physiology..

[35] Kolos E. A., Korzhevskii D. E. (2014). Neuroscience and Behavioral Physiology..

[36] Gill S.K., Ishak M., Rylett R.J. (2005). J Neurosci Methods..

[37] Korzhevskii D.E., Gilyarov A. V. (2010). Neuroscience and Behavioral Physiology..

[38] Giliarov A.V., Kirik O.V., Korzhevskii D.E. (2010). Morfologiia..

[39] Kirik O.V., Sukhorukova E.G., Vlasov T.D., Korzhevskii D.E. (2009). Morfologiia..

[40] Korzhevskii D.E., Kirik O.V., Baisa A.E., Vlasov T.D. (2009). Bull Exp Biol Med..

[41] Tippett L.J., Waldvogel H.J., Thomas S.J., Hogg V.M., van Roon-Mom W., Synek B.J., Graybiel A.M., Faull R.L. (2007). Brain..

[42] Korzhevskii D. E., Khozhai L.I., Gilerovich E. G., Grigoriev I. P., Gilyarov A. V., Otellin V.A. (2006). Proceedings of the All-Russian conference with international participation. Structural, functional and neurochemical regularities of the brain asymmetry and plasticity-2006., Moscow..

[43] Igarashi T., Huang T.T., Noble L.J. (2001). Exp Neurol..

[44] Davoli M.A., Fourtounis J., Tam J., Xanthoudakis S., Nicholson D., Robertson G.S., Ng G.Y., Xu D. (2002). Neuroscience..

[45] Unal-Cevik I., Kilinç M., Gürsoy-Ozdemir Y., Gurer G., Dalkara T. (2004). Brain Res..

